# Bilateral thoracoscopic splanchnicectomy for pain in patients with chronic pancreatitis impairs adrenomedullary but not noradrenergic sympathetic function

**DOI:** 10.1007/s00464-012-2152-4

**Published:** 2012-03-07

**Authors:** H. C. J. L. Buscher, J. W. M. Lenders, O. H. G. Wilder-Smith, C. G. J. Sweep, H. van Goor

**Affiliations:** 1 Department of Surgery, Radboud University Nijmegen Medical Centre, P.O. Box 9100, 6500 HB Nijmegen, The Netherlands; 2 Section of Vascular Medicine, Department of Medicine, Radboud University Nijmegen Medical Centre, Nijmegen, The Netherlands; 3 Department of Anaesthesiology, Pain and Palliative Care, Radboud University Nijmegen Medical Centre, Nijmegen, The Netherlands; 4 Department of Laboratory Medicine, Radboud University Nijmegen Medical Centre, Nijmegen, The Netherlands; 5 Department of Internal Medicine III, University Hospital Carl Gustav Carus, Dresden, Germany

**Keywords:** Sympathetic activity, Splanchnicectomy, Thoracoscopy, Chronic pancreatitis

## Abstract

**Background:**

Bilateral thoracoscopic splanchnicectomy (BTS) is a well-known technique to alleviate intractable pain in patients with chronic pancreatitis. BTS not only disrupts afferent fibers from the pancreas that mediate pain but also postganglionic sympathetic fibers, which originate in segments T5–T12 and which innervate the vasculature of the liver, pancreas, and the adrenal gland. The purpose of this study was to assess whether and how BTS affects sympathetic noradrenergic and adrenomedullary function in patients with chronic pancreatitis.

**Methods:**

Sixteen patients with chronic pancreatitis for at least 1 year underwent autonomic function testing before and 6 weeks after BTS for intractable pain. Testing was performed during supine rest and during sympathetic stimulation when standing.

**Results:**

Supine and standing systolic and diastolic blood pressure were significantly lower post-BTS compared with pre-BTS (*P* = 0.001). One patient showed orthostatic hypotension after BTS. Baseline plasma norepinephrine levels and plasma norepinephrine responses to sympathetic activation during standing were not reduced by BTS. In contrast, supine plasma epinephrine levels and responses during standing were significantly reduced (*P* < 0.001). Parasympathetic activity was unaffected by BTS as shown by unaltered Valsalva ratio, I-E difference, and ΔHRmax.

**Conclusions:**

BTS for pain relief in patients with chronic pancreatitis reduced adrenomedullary function, due to disruption of the efferent sympathetic fibers to the adrenal gland. BTS did not affect noradrenergic sympathetic activity, although blood pressure was lower after the sympathectomy.

Chronic pancreatitis is a fibroinflammatory disease of the pancreas. Pain is the main reason for these patients to seek medical help. The goal of surgical therapy is to alleviate pain, preferably without morbidity or mortality and with preservation of pancreatic function. In the absence of an anatomical substrate or when previous surgery has not been successful, nerve interruption, such as celiac blockade or splanchnicectomy, are recommended [1]. Bilateral thoracoscopic splanchnicectomy (BTS) is a minimally invasive surgical procedure to dissect splanchnic nerves through a thoracoscopic approach. BTS has the advantage of low morbidity without reported mortality [2].

The splanchnic nerves predominantly consist of sympathetic fibers and the contribution of visceral pain afferents, which accompany the sympathetic nerves is relatively small [3]. Sympathetic fibers, originating in segments T5–T12, synapse in the celiac ganglia from where postganglionic splanchnic fibers innervate the pancreatic and intestinal vasculature [4] and the adrenal glands [5]. A potentially undesired effect of BTS is an impairment of cardiovascular homeostasis, particularly in chronic pancreatitis patients who often have cardiovascular and respiratory comorbidity. Because the splanchnic vascular bed has an important capacitance function, interrupting its noradrenergic innervation by BTS might reduce the return of venous blood to the heart, thus predisposing the patient to orthostatic hypotension. In addition, dissecting the sympathetic nerve to the adrenal gland might affect adrenomedullary function. This study was designed to assess the effects of BTS performed in patients with chronic pancreatitis on sympathetic noradrenergic and adrenal medullary baseline function before and after sympathetic stimulation.

## Patients and methods

Sixteen patients (10 men and 6 women), with chronic pancreatitis for at least 1 year, who underwent bilateral thoracoscopic splanchnicectomy (BTS) for disabling pain were studied for the effects of sympathectomy on sympathetic activity. Median age was 49 (range 32–68) years. All patients had chronic pancreatitis, diagnosed by means of endoscopic retrograde cholangiopancreaticography, computed tomography, and/or magnetic resonance cholangiopancreaticography, according to standard criteria [6]. Based on these criteria, all but three patients had small-duct disease. Fifteen patients were taking opioid medication. Patients treated for hypertension or patients with medications that directly interfered with the sympathetic nervous system were excluded. Demographic data and medication are listed in Table 1. All patients gave their written, informed consent for this study. The medical ethical committee waved the need for protocol review.

**Table 1:** Demographic data and medication of the pancreatitis patient

Demographics	
Patients	16
Sex (M/F)	10/6
Age (years), median (range)	49 (32–68)
Onset of pancreatitis, median (range)	5.5 (1–21.7)
Etiology	
Alcohol	7
Idiopathic	7
Other (hereditary, hypertriglyceridemia-induced pancreatitis)	2
Use of opioids	15
Morphine equivalents (mg), mean (range)^a^	146 (0–520)
Use of sedatives	2
Diabetes mellitus	4

^a^Opioid medication has been converted to Morphine Equivalents (MEq) using the Narcotic Analgesic Converter (Version 2.0, GlobalRPh^©^ Inc.; www.GlobalRPh.com)

### Splanchnic denervation

We used a technique for BTS that was slightly modified from that described by Cuschieri et al. [7]. In summary, the procedure is performed under general anesthesia with double-lumen intubation for single-lung ventilation. The patient is placed in a full prone position with the arms stretched along the head. The thoracoscope is introduced two intercostal spaces below the angle of the scapula, and a second trocar is introduced one intercostal space above and a few inches toward the spine. The parietal pleural space is opened from the 5th to the 12th thoracic vertebra. To ensure complete denervation, the splanchnic nerves and all of the potentially nerve-bearing tissue on each side as well as the sympathetic chain itself at level T5 are carefully transected by using the harmonic scalpel (Ultracision^®^, Johnson & Johnson Medicals, Amersfoort, The Netherlands). At the end of the procedure, the lungs are reexpanded and the trocar sites closed without routine use of chest drains.

### Autonomic function testing

Sedative medication, which remained unaltered after the operation, was stopped the day before testing, while analgesic treatment was continued. All patients underwent noninvasive measurement of blood pressure and heart rate by the Finapres device (TNO, The Netherlands, model 5) before and 6 weeks after BTS [8]. Blood pressure and heart rate were measured after supine rest for 20 min. Thereafter, the following tests for autonomic function were performed:

*Standing up maneuver*. Patients were asked to stand up within 5 s and to remain in the upright position for 2 min. The systolic (SBP) and diastolic blood pressure (DBP) responses to the upright position were calculated from the blood pressure values 50–80 s after standing up compared with those of the supine rest position. Orthostatic hypotension was defined as a decrease of systolic blood pressure of more than 20 mmHg after standing. The difference between the maximal heart rate at standing (15–20 s after standing up) and the supine heart rate was calculated and expressed as ∆HRmax. The quotient between maximal heart rate and minimal heart rate after standing up was calculated as T/B ratio (tachycardia/bradycardia ratio) [9].

*Forced breathing*. Each patient performed six maximal respirations in 1 minute in the supine position, and both inspiration and expiration were synchronized with the program clock on the computer. Heart rate was computed from the RR-interval and the difference between maximal and minimal heart rate during each cycle of 10 seconds was measured and averaged for the six respiratory cycles (I-E difference) [9].

*Valsalva maneuver*. All patients were instructed to maintain an expiratory pressure of 40 mmHg for 15 s by means of forced expiration into a mouthpiece connected to a manometer. The maneuver was performed in triplicate. The maximum heart rate during the maneuver was divided by the minimum heart rate after the manoeuvre (Valsalva ratio) [10].

### Plasma catecholamines

After voiding, a cannula was inserted in an antecubital vein and a blood sample was drawn after 20 min of supine rest. To assess the effects of mild sympathetic stimulation, a second blood sample was drawn after 5 min of standing. Blood samples were collected into chilled tubes containing heparin and were immediately placed on ice. All samples were centrifuged (at 4°C) within 1 hour after blood sampling and plasma samples were stored at −80°C until assayed for concentrations of norepinephrine (NE) and epinephrine (EPI), using high-performance liquid chromatography [11]. All samples of each patient were assayed within the same run. Intra-assay coefficients of variation were 1.9% for NE and 3% for EPI. Interassay coefficients of variation were 6.5% for NE and 11.4% for EPI.

### Statistical analysis

For all variables, nonparametric tests were used. Preoperative and postoperative data of the entire group were compared using the paired Wilcoxon signed rank-sum test. Relative and absolute differences between baseline and standing NE and E levels (Δ) were calculated. We performed statistical analysis using SPSS^®^ for Windows (Release 13.0, SPSS Inc., Chicago, IL, USA). Demographic data are presented as median and range, and all other data are presented as medians and interquartiles (IQR). Differences were considered statistically significant if *P* < 0.05.

## Results

In all 16 patients, BTS was technically successful, meaning that all potentially nerve-bearing tissue on each side had successfully been transected.

### Hemodynamic effects of BTS

After BTS, supine as well as standing, both median systolic and diastolic blood pressures were significantly lower than before BTS (*P* < 0.01; Table 2). This effect was observed in all but two patients. The median supine systolic and diastolic blood pressures were 10.5 mmHg (IQR 4–15.8 mmHg) and 7.5 mmHg (IQR 3–10.5 mmHg) lower postoperatively. During standing the median differences were 10.5 mmHg (IQR 7–27 mmHg) and 7.5 mmHg (IQR 1–17 mmHg), respectively. DBP increased significantly with standing both before and after the operation (2.5 mmHg (IQR −1 to 11 mmHg), *P* = 0.012, and 3 mmHg (IQR −1 to 7 mmHg, P = 0.049), whereas SBP did not. One patient demonstrated orthostatic hypotension before BTS, which persisted after the procedure. One patient who had no orthostatic hypotension before BTS demonstrated asymptomatic orthostatic hypotension after BTS. Heart rate showed a similar increase with standing both before and after BTS.

**Table 2:** Results of the autonomic function testing pre and postoperatively, depicted as median (IQR)

	Pre-BTS	*P* ^b^	Post-BTS	*P* ^a^	*P* ^c^
Valsalva ratio	1.26 (1.13–1.54)		1.22 (1.17–1.88)	0.45	
IE_difference	10 (8.4–17.1)		12.0 (6.7–15.8)	0.701	
∆ HRmax	22 (16–31)		18 (16–25)	0.308	
T/B ratio	1.11 (1.07–1.23)		1.1 (1.06–1.16)	0.181	
SBP supine (mmHg)	108 (100–120)	0.856	102 (91–115)	0.028	0.074
SBP standing (mmHg)	109 (95–124)		97 (82–108)	0.01	
DBP supine (mmHg)	63 (50–72)	0.012	57 (51–65)	0.005	0.016
DBP standing (mmHg)	67 (59–79)		60 (48–72)	0.016	
MAP supine (mmHg)	78 (69–86)		72 (65–81)	0.009	
MAP standing (mmHg)	78 (74–95)		71 (60–83)	0.013	
HR supine (beats/min)	66 (62–75)	<0.001	67 (61–75)	0.477	0.001
HR standing (beats/min)	83 (70–97)^a^		89 (71–103)	0.026	

*P* ^a^ P post BTS vs. pre-BTS, *P* ^*b*^ P standing vs. supine preoperatively and *P* ^*c*^ P standing vs. supine postoperatively

### Autonomic function tests

In all patients, the Valsalva ratio, the I-E difference, and the ΔHR max were within normal limits before BTS and were unaffected by BTS. The postoperative T/B ratio did not differ significantly from the preoperative ratio (Table 2).

### Plasma catecholamines

In two patients, both plasma EPI and NE levels after BTS could not be measured for technical reasons.

#### Plasma NE levels

**Fig. 1:**
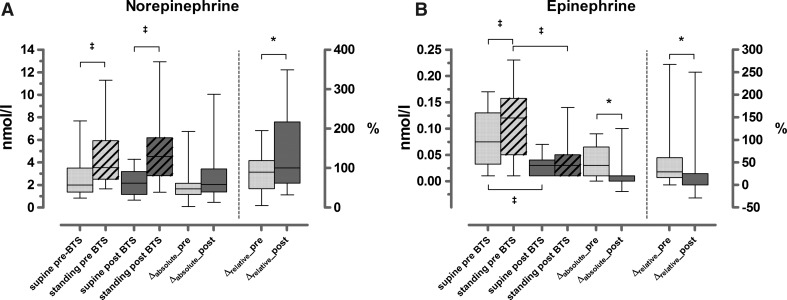
Boxplots of plasma norepinephrine (NE; **A**) and plasma epinephrine (EPI; **B**) levels before (*light boxes*) and after (*dark boxes*) the BTS. Δ_absolute_ is the absolute increment of NE or EPI after standing (pre = pre-BTS; post = post-BTS). On the right *Y*-axis, the relative increment Δ_relative_ is depicted (%, right *Y*-axis) (**A**, ^‡^ *P* = 0.001, *P = 0.011; **B**, ^‡^ *P* = 0.001, *P = 0.002, Wilcoxon signed rank-sum test). *Error bars* represent range (minimum–maximum)

Before BTS, five patients showed higher supine plasma NE levels than normal (upper limit NE 3.0 nmol/L), ranging from 3.06–7.68 nmol/L. During standing, the plasma NE levels of the entire group increased significantly from a median 2.0 nmol/L (IQR 1.37–3.49) to a median 3.53 nmol/L (IQR 2.52–5.94; *P* = 0.001). The median relative increment in plasma NE was 89% (range 48–119%; Fig. 1A).

After BTS, baseline supine plasma NE level was 2.17 nmol/L (IQR 1.16–3.18), which was not significantly different from the pre-BTS plasma NE level (*P* = 0.46). In the upright position, plasma NE rose to 4.52 nmol/L (IQR 2.82–6.19; *P* < 0.001), and the median relative increment was slightly but significantly higher than before BTS (100% vs. 89%; *P* = 0.011; Fig. 1B).

#### Plasma epinephrine levels

Before BTS, all patients had supine plasma EPI levels that were within the normal range (median 0.08 (IQR 0.04–0.13) nmol/L). During standing plasma EPI levels rose to median 0.12 nmol/L (IQR 0.06–0.16; *P* < 0.001). The relative increment of plasma EPI was 37% (IQR 21–66%; Fig. 1B).

After BTS, median supine plasma EPI level was 0.03 nmol/L (IQR 0.01–0.04) and significantly lower than before BTS (*P* < 0.001). After BTS, plasma EPI levels did not increase after standing. In each individual patient, both the supine and the standing plasma EPI levels were lower after BTS. Consequently, both the relative and the absolute increments in plasma EPI were significantly reduced after BTS (*P* = 0.002 and *P* = 0.001, respectively; Fig. 1B).

## Discussion

Interruption of sympathetic nerves by BTS for pain relief in patients with chronic pancreatitis reduces baseline and stimulated EPI plasma levels. In contrast, there is no effect on sympathetic noradrenergic and parasympathetic function. Furthermore, the blood pressure was significantly reduced after BTS.

Because the sympathetic nerves to the adrenal gland are interrupted by BTS and because the adrenal medulla is almost the sole source of circulating EPI, it is not surprising that adrenomedullary function is impaired, which is reflected by the decrease in plasma EPI levels. This effect has been shown before in animal studies and for the first time now in human subjects [12]. The present study cannot answer the question of whether this selectively impaired adrenomedullary function is of (patho-) physiological significance, because we did not examine the plasma EPI responses, for example, to strong stimuli, such as hypoglycemia, in these patients. However, from animal studies it is known that insulin-induced hypoglycemia detected in the portal vein is mediated through spinal sympathetic afferents and subsequently suppressed by sympathectomy [13, 14]. Theoretically, sympathectomy might have consequences for the detection and regulation of glucose metabolism in diabetic patients. In our diabetic patients, we did not encounter such problems after BTS. Furthermore, we know from adrenalectomized patients that depletion of EPI is tolerated well [15, 16]. Although the adrenal cortex also is sympathetically innervated, the main and predominant regulator of adrenal cortical function is ACTH, released by the anterior pituitary gland. Therefore, it is unlikely that adrenal cortical function is impaired after BTS.

Noradrenergic sympathetic nerves supplying the splanchnic vascular bed also are disrupted by BTS, but plasma NE levels were unaltered, suggesting that overall sympathetic nervous function is unimpaired. Although the splanchnic vascular bed is richly innervated by sympathetic nerves and the hepatic-mesenteric spillover amounts to approximately 40%, a relatively low fraction of circulating plasma NE is coming from the mesenteric organs, such as pancreas and liver [17]. In addition, circulating plasma NE is derived from sympathetic nerves from other organs, such as skeletal muscle and heart; therefore, it is possible that the local effect of BTS on splanchnic derived NE is obscured. To uncover this local effect, more selective and invasive measurements of NE spillover of the splanchnic area would be necessary.

Variable effects on blood pressure have been reported after thoracic sympathectomy. In contrast to Noppen et al. [18], who found no effect, Kingma et al. [19] and Papa et al. [20] demonstrated a decrease in resting BP after sympathectomy as we did. From a physiological point of view, the reduction can be attributed to vasodilatation due to a reduced vascular resistance [19] or to a decrease in cardiac output due to a decreased venous return. The lack of a reflex-mediated increase in heart rate and plasma catecholamines after BTS argues against the possibility of substantial vasodilation. A direct cardiovascular effect following denervation does not explain the hypotension in our patients, because the majority of the cardiac sympathetic plexus receives cardiac branches from above T5. In patients who undergo sympathectomy for hyperhidrosis, ganglia are transected at the levels T2–T4 through which the direct sympathetic nerves serve the heart and more cardiovascular effects are expected than after BTS [18–22].

Increased renal sympathetic activity causes hypertension and sympathectomy is applied for the treatment of hypertension [23–25]. Because the renal plexus receives fibers from the lesser splanchnic nerves and the celiac plexus, disruption of this anatomical and functional relation also might explain the hypotensive effect of splanchnicectomy.

Only one patient demonstrated orthostatic hypotension after BTS but was asymptomatic. Clinical significant orthostatic hypotension has only been reported in a minority of patients after both bilateral and unilateral thoracoscopic splanchnicectomy for pain in chronic pancreatitis [26, 27].

Reduction of pain may be held responsible for lowering blood pressure. Stress caused by pain causes an increase in blood pressure, by increasing the muscle sympathetic nerve activity [28]. However, the relationship of chronic pain and blood pressure is less well understood and from some studies it appears that this normal “pain–blood pressure” relationship is absent or reversed in patients with chronic pain [29, 30]. This is supported by the fact that all our patients were normotensive. Therefore, it is unlikely that reduction in pain is responsible for causing orthostasis or reduction in blood pressure in our group of patients. Because variable effects of opioids on blood pressure (increase as well as decrease) in normotensive as well as hypertensive state have been reported, opioid use may have affected the blood pressure in our patients [31, 32]. However, the opioid dose (expressed in MEq) was too diverse in our patients to explain the unequivocal blood pressure reduction measured in the majority of the patients.

Bilateral thoracoscopic splanchnicectomy is a surgical procedure in which the splanchnic nerves are dissected by a thoracoscopic approach. After 6 months follow-up 60–75% of the patients have pain relief [33]. Because at least 25% of patients do not experience any pain relief at all after splanchnic deafferentation of the pancreas from the beginning, complete splanchnic transsection could be criticized in these patients. The finding of low EPI levels in our cohort of patients in rest as well as after stimulation post BTS, suggests complete technical success. Measuring EPI concentrations before and after BTS therefore might be a tool to verify complete denervation.

Bilateral splanchnicectomy does not seem to have significant clinical side-effects. However, some caution is warranted regarding the hypotensive effect, especially in younger patients with a normal to low preoperative blood pressure. In patients with hypertension, BTS may necessitate adjustments in the antihypertensive treatment.
